# Combinations of targeted therapies in human cancers

**DOI:** 10.18632/aging.101085

**Published:** 2016-10-26

**Authors:** Didier Decaudin, Christophe Le Tourneau

**Affiliations:** Institut Curie, 75248 Paris, France

**Keywords:** targeted therapies, human cancers, preclinical studies, clinical trials

Few human cancers are driven by one specific molecular event, such as, for example, chronic myeloid leukemia. Human cancers appear to be generally derived from multiple oncogenic alterations maturing through their own particular mechanisms [1]. One major consequence of such alterations is the dysregulation of intracellular signaling pathways favoring tumor cell survival, proliferation, and motility. These altered signaling pathways may be hierarchically organized in untreated tumors and therefore allow the identification of the single most relevant target. The recent identification of driver oncogenic events has led to the development of targeted therapies that have dramatically improved the prognosis of cancer patients for some tumor types treated with the matched targeted therapy, such as in *BRAF*^V600E^-mutant cutaneous melanoma and translocated-ALK lung adenocarcinomas. However, this only applies to a small proportion of patients, since not all cancers harbor a druggable molecular alteration. In addition, it has not been demonstrated that targeted therapy should be given solely on the basis of the tumor molecular profile [2]. Finally, resistance to targeted therapy always occurs eventually. Resistance to targeted therapy often involves the appearance of new molecular alterations, as reported in *BRAF*^V600E^-mutant cutaneous melanoma patients treated with a BRAF inhibitor [3]. One way to overcome resistance to single-agent targeted therapy may be the use of drug combinations, such as trastuzumab and lapatinib in Human Epidermal Growth Factor Receptor 1- and/or 2-positive metastatic breast cancer [4], or dual BRAF and MEK blockade in *BRAF*^V600^-mutant melanoma [5]. We believe that the future of targeted therapy relies on combinations, but key preclinical and clinical criteria must be met before moving a new targeted therapy combination into clinical evaluation.

Three major aspects should be carefully evaluated in preclinical studies: (i) The treatment combination should display a significantly higher antitumor activity than each compound as a single agent. Results should consistently be observed in several animal models. Solutions such as the “single-mouse” schedule have been proposed to reduce the number of treated animals [6]. This latter approach allows powerful statistical assessments, but raises methodological issues due to the *in vivo* variability of tumor growth. (ii) The treatment combination should induce tumor shrinkage and not only decrease the speed of tumor growth, even if statistically significant. Indeed, a reduction of tumor volume reflects an effect on both tumor cell proliferation and tumor cell survival with the possible induction of apoptosis, better predicting the clinical efficacy of the combination treatment. (iii) Synergy should be demonstrated between the tested compounds, since it represents the most frequent formulated paradigm used for the selection of relevant treatment combinations after *in vitro* determination. Synergy may reflect molecular crosstalk between targeted signaling pathways which may increase the efficacy of the therapies. However, there is sometimes no *in vitro* synergy, although it was reported *in vivo*. For example, no *in vitro* synergy was observed for a combination of a protein kinase C inhibitor and a p53-MDM2 inhibitor in various uveal melanoma cell lines, whereas strong *in vivo* efficacy of the combination was observed in patient-derived xenografts relative to each compound given alone, probably due to p53-MDM2-induced apoptosis [7]. We therefore encourage *in vivo* experimentation in addition to *in vitro* screening, especially in cases of disappointing *in vitro* evaluation, despite a strong biological rationale.

While these preclinical aspects are key, clinical considerations are also important before moving into the clinic. Three main clinical aspects should also be discussed before launching a combination clinical trial: (i) The treatment combination should act on different signaling pathways that have been demonstrated to be dysregulated in cancer patients. This point remains difficult to appreciate due to the uncertain crosstalk between molecular alterations identified through large-scale genomic testing [2]. The identification of predictive factors for each compound of the combination may increase the likelihood of success, along with the selection of targets defined as prognostic factors in the outcome of patients treated with each compound. (ii) The treatment combination should be clinically feasible in terms of administration routes and sequences, in particular due to pharmacokinetic interactions such as cytochrome activity modulation. Pharmacokinetic interactions between agents may hamper the development of certain drug combinations. A careful review of the metabolism of the drugs is necessary to carefully implement the dose-finding clinical trial of the drug combination. (iii) The occurrence of overlapping toxicities should be anticipated and also taken into account when designing the dose escalation in the dose-finding trial. Preclinical models poorly predict drug tolerance in humans and may not be very helpful [8], even in the presence of well-demonstrated synergy. Drug combination efficacy may also not be maintained if lower doses of drugs have to be used.

**Figure 1:**
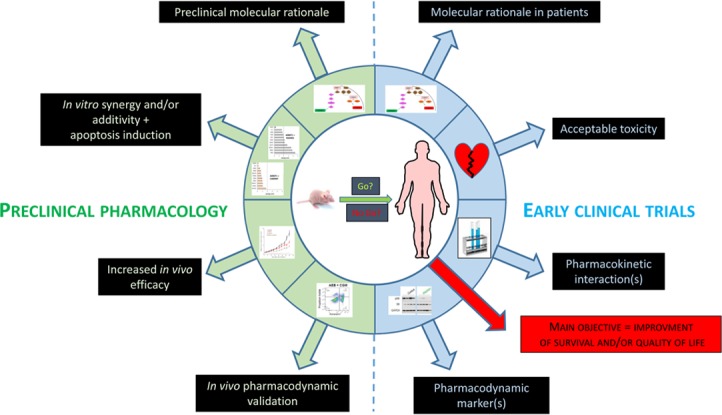
Preclinical and clinical criteria for the validation of combinations of targeted therapies in cancer patients.

In conclusion, we believe that these primary preclinical and clinical criteria should all be carefully assessed before each clinical study to evaluate targeted treatment combinations (Figure 1). The criteria detailed above are not exhaustive, but should be sufficient to help avoid wasting money and jeopardizing patient safety if they are carefully evaluated before moving a targeted therapy combination into the clinic.
